# MALICoT: A pilot cross‐sectional study analyzing the effects of power training and age on endomysium content and fiber area in the soleus muscle of adult males

**DOI:** 10.14814/phy2.70933

**Published:** 2026-05-29

**Authors:** Christoph S. Clemen, Sebastian W. Humbsch, Carolin Berwanger, Leonid Mill, Andreas Schmidt, Rolf Schröder, Jörn Rittweger

**Affiliations:** ^1^ Institute of Aerospace Medicine German Aerospace Centre (DLR) Cologne Germany; ^2^ Institute of Vegetative Physiology, Medical Faculty University of Cologne Cologne Germany; ^3^ University Hospital for Pediatrics and Adolescent Medicine, Faculty of Medicine University of Cologne Cologne Germany; ^4^ MIRA Vision Microscopy GmbH Göppingen Germany; ^5^ Center for Molecular Medicine Cologne (CMMC), Medical Faculty, University of Cologne and Cologne Excellence Cluster on Cellular Stress Responses in Aging‐Associated Diseases (CECAD) University of Cologne Cologne Germany; ^6^ Institute of Neuropathology, University Hospital Erlangen Friedrich‐Alexander University Erlangen‐Nürnberg Erlangen Germany; ^7^ Present address: Bruker Daltonics GmbH & Co KG Bremen Germany

**Keywords:** aging, deep learning‐based image analysis, endomysium, masters athletes, muscle exercise, myopathology

## Abstract

The cross‐sectional Master Athletic Laboratory Study of Intramuscular Connective Tissue (MALICoT, DRKS00015764) examined effects of athletic exercise and age on endomysium content in human soleus muscle. Forty‐three clinically healthy male participants were grouped into young (20–35 years) nonphysically active controls (*n* = 12), young power‐trained athletes (*n* = 10), older (60–75 years) nonphysically active controls (*n* = 11), and older power‐trained athletes (*n* = 10). Biopsy specimens from the left soleus muscle of each participant were cryo‐sectioned for (i) routine histological staining for myopathological evaluation, (ii) deep learning‐based image analysis of H&E‐ and MHC‐stained sections, (iii) laminin‐γ‐1/collagen IV, collagen I, and collagen III immunofluorescence image analysis, and (iv) quantitative proteomics. Myopathological evaluation revealed normal skeletal muscle in 26 participants, while 11, 4, 1 and 1 biopsies showed unspecific myopathological changes, chronic neurogenic atrophy, type‐II fiber atrophy, and unspecific myositic changes, respectively. Analysis of H&E‐, MHC‐, laminin‐, and collagen‐stained sections revealed a 1.3‐fold higher level of “mean fiber area” in young power‐trained and aged unathletic participants. No significant effect was detected in the endomysium content. Proteomic analysis revealed no group‐specific differences except that plasma membrane calcium‐transporting ATPase 2 was less abundant in samples from aged power‐trained athletes. Overall, neither athletic exercise nor age had an effect on the content or composition of the endomysium in the soleus muscle of male humans.

## 1 INTRODUCTION

Skeletal muscle function declines with age, leading to a reduction in muscle mass and mechanical output. Although numerous studies have investigated this decline in muscle mass and function (Larsson et al., 2019), the intramuscular connective tissue has received little attention. The endomysium and perimysium are generally thought to play a mechanical role, both in distributing mechanical stresses and allowing slippage between fibers and between fascicles (Purslow, 2020). It has been assumed that aging is associated with the accumulation of intramuscular connective tissue in mice (Fede et al., 2022; Yeung et al., 2023), rats (Alnaqeeb et al., 1984), and humans (Fede et al., 2022). We previously reported that the endomysium content in the soleus muscles of participants increased in response to 60 days of experimental bed rest when normalized to muscle fiber area, but not when normalized to muscle fiber number (Thot et al., 2021), suggesting that the total intramuscular connective tissue content remains constant during at least 2 months of immobilization. Thus, it is possible that the reported accumulation of intramuscular connective tissue with age is more an effect of fiber shrinkage rather than a true increase in intramuscular connective tissue content.

In the cross‐sectional Master Athletic Laboratory Study of Intramuscular Connective Tissue (MALICoT), which was conducted as a complementary study to the research on bed rest‐induced muscle wasting (Thot et al., 2021), we addressed the hypothesis that both age and training status would have an effect on the endomysium content in the soleus muscle. Since aging is typically associated with a more sedentary lifestyle (Varo et al., 2003), so that the muscle phenotype observed in older individuals is influenced by both senescence and immobilization, we focused on Masters athletes. These athletes continue to train and compete in sport at older ages, often well into their 8th or 9th decade of life (Tanaka et al., 2019). We further focused on the soleus muscle, which acts as a spring and supporter muscle during running (Hamner et al., 2010; Lai et al., 2019; Pandy et al., 2021), and selected athletes specialized in sprinting and jumping, as elastic energy storage in the calf muscles is paramount in these activities (Cavagna et al., 2008). We assumed that the mechanical demands on the intramuscular connective tissue would be greatest during spring action. Due to the intrinsic difficulty of organizing a longitudinal, interventional study in healthy human volunteers, we chose a fully factorial cross‐sectional approach comparing four groups of athletes and nonathletes at young and old ages.

## 2 MATERIALS AND METHODS

### 2.1 Ethical approval and study design

The Master Athletic Laboratory Study of Intramuscular Connective Tissue (MALICoT) was approved by the Ethics Committee of the North Rhine Medical Association, Düsseldorf, Germany, reference number 2018269, and was registered in the German Clinical Trials Register, DRKS‐ID DRKS00015764. All participants gave written informed consent before participating in the study. The study adhered to the principles of the Declaration of Helsinki and to the General Data Protection Regulation (GDPR). A total of 43 clinically healthy male participants with a body mass index ≤28 kg/m^2^ could be included in the study and they were tested between August 2020 and May 2021. Blinded to the investigators, the participants were divided into four different groups: young nonathletes (*n* = 12) and young power‐trained athletes (*n* = 10) (age range 20 to 35 years) and old nonathletes (*n* = 11) and old power‐trained athletes (*n* = 10) (age range 60 to 75 years). The participants completed a questionnaire to quantify their exercise‐related metabolic equivalents for task (METs) (Frey et al., 1999), underwent medical examination, and anthropometric data were collected. Participants who did at least 4 h of running training per week, which was also monitored by means of an accelerometric recording box worn for at least 1 week, and participated in running competitions were categorized as athletes. The running training of these participants also included sprinting and jumping exercises. Participants were categorized as unathletic if they had little or no sports‐related physical activity, defined by an activity‐related energy expenditure of ≤2 METs. Further information on MALICoT has been published, including participants characteristics and biomechanical parameters (Sanchez‐Trigo et al., 2022), bone geometry data (Scorcelletti et al., 2023), physical activity and cardiometabolic risk profiling data (Rittweger et al., 2024), and muscle volume, fat fraction and power data (Zange et al., 2026).

### 2.2 Soleus muscle biopsy

Biopsies were taken from the left soleus muscle of all participants under local anesthesia and in sterile conditions using a vacuum‐assisted biopsy system with a 10G needle (Vacora Biopsy System type VF2019, Bard) in an ultrasound‐assisted lateral approach. This yielded cylindrical muscle tissue specimens that were largely mechanically intact, measuring approximately 19 mm in length, 3 mm in diameter and 150 mg in weight. The obtained biopsy tissue cylinders contained no muscle fascia material. The physical principle of this automated biopsy system with single‐use biopsy needles is similar to the manual Bergström biopsy technique, but with inverted tubes (the outer one is mobile) and with much less mechanical damage to the biopsy specimen (Akarolo‐Anthony et al., 2012; Lee et al., 2020). Part of the tissue cylinders were mounted on cork plates (15 mm diameter, 3 mm thickness) with fiber orientation for transverse cryosectioning, covered with Tissue‐Tek OCT Compound (Sakura Finetek, Torrance, CA, USA), snap‐frozen in liquid nitrogen‐cooled isopentane to avoid freezing artifacts, collected in 5 mL LDPE sample vials with caps, and stored at −80°C for subsequent analyses.

### 2.3 Histological staining

Cross‐sections of 6 μm thickness of the soleus muscle biopsy specimens were prepared with a cryotome (Leica CM 1850 UV), collected on microscope slides, and used for routine histology protocols (Dubowitz et al., 2013). The routine histochemistry stains comprised H&E, Oil red O, Gömöri trichrome, COX/SDH, and PAS, and the immunohistochemistry stains comprised slow MHC (MHCs), fast MHC (MHCf), developmental MHC (MHCd), and neonatal MHC (MHCn) staining. Antibodies used for immunohistochemistry were mouse monoclonal anti‐myosin heavy chain slow (Leica Biosystems/Novocastra, NCL‐MHCs, 1:50), mouse monoclonal anti‐myosin heavy chain fast (Leica Biosystems/Novocastra, NCL‐MHCf, 1:20), mouse monoclonal anti‐myosin heavy chain developmental (Leica Biosystems/Novocastra, NCL‐MHCd, 1:20), and mouse monoclonal anti‐myosin heavy chain neonatal (Leica Biosystems/Novocastra, NCLß‐MHCn, 1:10).

### 2.4 Myopathological evaluation

A thorough histopathological evaluation was carried out by an expert myopathologist, who is one of the authors of this manuscript, an internationally recognized researcher in the field of neuromuscular disorders, a member of the German Reference Center for Neuromuscular Diseases of the German Society for Neuroanatomy and Neuropathology since 2008, and whose excellent expertise is very well documented in numerous publications (representative publications with special focus on skeletal muscle pathology (Argente‐Escrig et al., 2021; Clemen et al., 2015; Fischer et al., 2003; Hübbers et al., 2007; Kiphuth et al., 2010; Schröder et al., 2000; Schröder et al., 2002; Schröder et al., 2003; Schröder & Schoser, 2009; Türk et al., 2014)). The criteria and thresholds for the myopathological diagnoses were as follows: Chronic neurogenic atrophy was defined by the presence of at least one type I and one type II fiber group, each containing at least 15 fibers of the respective fiber type, optionally in conjunction with atrophic fibers and nuclear clumps. Type II fiber atrophy was defined by a large number (>50%) of type II fibers with a fiber diameter that was >12% smaller than that of type I fibers. Unspecific myositic changes in one of the samples were defined by a small endomysial and small perimysial inflammatory infiltrate, which are never present in normal skeletal muscle. However, the diagnostic criteria for dermatomyositis, polymyositis, immunomediated necrotizing myopathy and inclusion body myositis were not fulfilled. Unspecific myopathological changes were defined by minor pathological alterations, such as combinations of sporadic atrophic type I or type II fibers, the occasional rounding of fibers, or insignificant numbers of ragged‐red or COX‐negative fibers, which precluded the diagnosis of normal skeletal muscle.

### 2.5 Parameter acquisition using an automated deep learning‐based biomedical image approach

Whole slide images of the H&E‐, MHCs‐ and MHCf‐stained soleus muscle sections (typically three sections per slide) were recorded by a slide scanner (NanoZoomer S60, Hamamatsu, Japan) and subjected to an automated deep learning‐based image analysis software tool based on photorealistic, computer graphics generated synthetic data in muscle histopathology (Mill et al., 2025). In this study, the software was used to identify the stained muscle tissue sections, transverse muscle fiber orientation, sectioning and staining artifacts, and to segment muscle fibers (H&E, MHCs and MHCf stains) and endomysium (H&E stain only). For H&E‐stains, the following parameters were obtained for each muscle fiber, each manually added region of interest (ROI), which typically contained no perimysium and about 100 muscle fibers (sufficient for reliable quantification (McCall et al., 1998)), each whole muscle tissue section, and each full slide, and output as individual, total or mean values: “number of samples,” “tissue area,” “number of fibers,” “fiber area,” “fiber area/tissue area,” “fiber diameter,” “fiber perimeter,” “endomysium area,” “endomysium area/tissue area,” “endomysium thickness,” “endomysium area/fiber area,” and “endomysium area/fiber number.” Note that setting a ROI in this software tool does not result in fibers being trimmed at the edges of a ROI, but that they are included in their entirety. In the case of MHCs‐ and MHCf‐stains, for each muscle fiber, each whole muscle tissue section and each full slide, the individual, total or mean values “number of samples,” “number of fibers,” “MHC‐staining positivity,” “fiber area,” and “fiber diameter” were determined.

### 2.6 Indirect immunofluorescence staining

Cryostat sections of 6 μm thickness were collected on microscope slides, air‐dried for 30 min, fixed in formaldehyde prepared from 0.5% paraformaldehyde for 10 min, washed three times in PBS for 5 min, and blocked with 5% NGS/0.2% Tween‐20 in PBS for 30 min (laminin‐γ‐1/collagen IV double‐staining) or 10% NGS/0.2% Tween‐20/0.15% glycine for 3 h (collagen I and collagen III single staining). For staining of plasma membrane calcium‐transporting ATPase 2 (ATP2B2/PMCA2), dried sections were fixed in −20°C cold acetone for 10 min, washed, and blocked with 5% NGS/0.2% Tween‐20 in PBS. Blocked sections were incubated with the primary antibodies diluted in the respective blocking buffer at room temperature for 1 h, for laminin‐γ‐1/collagen IV double‐staining with rabbit polyclonal anti‐laminin‐γ‐1 (Immundiagnostik, AP1001.2, 1:200) and mouse monoclonal anti‐collagen IV (Abcam, AB86042, 1:100), for the collagen I and collagen III single stains with either mouse monoclonal anti‐collagen I (Sigma‐Aldrich, C2456, 1:100) or mouse monoclonal anti‐collagen III (Sigma‐Aldrich/Merck‐Millipore, MAB3392, 1:100), and for plasma membrane calcium‐transporting ATPase 2 with rabbit polyclonal anti‐PMCA2 ATPase (Invitrogen, PA1‐915, 1:200). After six washes in PBS for 10 min each, sections were incubated with the appropriate secondary antibodies diluted 1:500 in blocking solution, goat anti‐rabbit AlexaFluor568 (Invitrogen, A‐11011) and goat anti‐mouse AlexaFluor647 (Invitrogen, A‐21236) together with DAPI at 1:1000 dilution for 45 min; for PMCA2 goat anti‐rabbit AlexaFluor555 (Invitrogen, A‐21429) diluted 1:400. Final washes were three times with 0.5% Triton X‐100 in PBS and five times with PBS for 15 min each, before sections were rinsed in ddH_2_O and embedded in Mowiol/DABCO.

### 2.7 Parameter acquisition by microscope and image software and manual image analysis

Laminin and collagen immunofluorescence images were recorded using a Zeiss Axio Imager.Z2m microscope (Carl Zeiss Microscopy, Oberkochen, Germany) with a 40x oil objective (NA 1.4). The Zeiss Zen software version 3.4 was used to stitch together the tile images into full section overviews and to set a region of interest (ROI) for each tissue specimen for subsequent image analysis. A ROI typically contained 100 muscle fibers (sufficient for reliable quantification (McCall et al., 1998)) and was selected so that the muscle fibers had a clear transverse orientation in the section, without perimysium and without artifacts from cryosectioning or staining.

Based on a previously established protocol, in which the laminin‐γ‐1 stained basement membrane was used to segment muscle fibers using the Zeiss Zen software version 3.4 (Thot et al., 2021), here the fluorescence channels of laminin‐γ‐1 and collagen IV were merged, as this resulted in an improved segmentation pattern. The software‐derived parameters of muscle fibers were number of fibers, fiber area, minimal and maximal Feret‐diameters, fiber perimeter, and ROI dimensions. In addition, the number of trimmed fibers, i.e., the fibers at the edges of a ROI, was determined manually to correct the number of fibers by calculating the difference between the software‐derived total number of fibers minus half of the number of trimmed fibers. The mean fiber area was calculated as the software‐derived total fiber area, including the trimmed fibers, divided by the corrected number of fibers. As the endomysial space is delineated by the laminin‐γ‐1 and collagen IV stained basement membranes of adjacent muscle fibers, the endomysial parameters were calculated according to (Thot et al., 2021). The total endomysium area was the difference between the ROI area and the total fiber area, and the mean endomysium thickness adjusted for trimmed fiber effects was the total endomysium area divided by the difference of the half of the total fiber perimeter minus the ROI perimeter.

In the case of the analysis of the collagen I and collagen III stained muscle sections, in which the endomysial area is labeled, the respective ROI images and parameters were exported from the Zeiss Zen software, and the ROI images were imported for a pixel‐based analysis into the freeware IrfanView version 4.60 (https://www.irfanview.com/) to derive endomysial and muscle fiber parameters. Imported ROI images were converted to black and white images, and the numbers of all ROI and black endomysial and white muscle fiber area pixels were obtained as well as pixel dimensions to calculate areas. The total number of fibers and trimmed fibers were counted manually and used to calculate the corrected number of fibers and the mean fiber area. The mean endomysial diameter was calculated as above, considering muscle fibers as squares, so that the total fiber perimeter was estimated as being four times the square root of the mean fiber area multiplied by the corrected number of fibers.

### 2.8 Muscle parameters, statistical analysis, and miscellaneous methods

Four main muscle parameters were obtained or calculated for the purpose of this work using different image analysis approaches as described above, in order to determine the soleus muscle endomysium content as a main objective of MALICoT, and to simplify and improve approaches for quantitating muscle connective tissue. All data were collected in a structured manner in Excel tables (Excel 2016, Microsoft) and imported into GraphPad Prism version 10.5.0.774 (GraphPad Software, Boston, MA) to perform basic statistics, normality tests, unpaired two‐sample Welch's *t*‐tests or Mann–Whitney *U* tests, as appropriate, and scatter plots for data visualization. The number of samples and *p*‐values are given in the figures and figure legends. Graphs and microscopic and other images were further processed and figures were assembled using CorelDraw Graphics Suite X7 (Corel Corporation, Ottawa, Canada).

### 2.9 Proteotypic peptide‐based quantitative proteomic analysis

Due to the limited amount of human soleus muscle tissue in the biopsy samples, the cryo‐specimens were used for both histology and proteomics. During preparation of the blank cryo‐sections for histological analysis, an additional 10 cryo‐sections of 20 μm thickness were collected from each sample and dissolved in 10% SDS in PBS by boiling at 95°C for 10 min and subsequent sonication (30 s on, 30 s off) using a Bioruptor sonication bath at 14°C for 10 min. Disulfide bonds were reduced by addition of 5 mM DTT and incubation at 55°C for 30 min, and alkylated by addition of 40 mM chloroacetamide and incubation at room temperature for another 30 min. Samples were centrifuged at 20,000 x g for 10 min and 100 μL of the supernatant of each sample was transferred into a 96‐well plate PCR plate. 2.5 μL of magnetic SP3 protein affinity beads (a 1:1 mixture of the 1 μm Sera‐Mag SpeedBead carboxylate‐modified [E3] and [E7] magnetic particles, Cytiva, #65152105050250 and #45152105050250) and immediately 100 μL of acetonitrile were added to the samples. Proteins were allowed to bind for 10 min, followed by washing with 70% ethanol (3× 100 μL) and 100% acetonitrile (100 μL). After drying the beads, proteins were digested with 0.5 μg trypsin in 50 mM ammonium bicarbonate at 35°C for 12 h. Subsequently, peptide mixtures were desalted on SDB‐RP stage tips, vacuum dried and redissolved in LC–MS loading buffer (5% FA, 2% acetonitrile in water).

For LC–MS analysis, 300 ng of peptides were separated using a nanoElute HPLC chromatography system with a 5 mm trapping column and a 25 cm separation column. A linear gradient from 5 to 35% acetonitrile was applied over a time of 35 min to fractionate the peptide sample (pulled tip, 300 nL/min, 50 min). Eluting peptides were directly ionized and detected in a timsTOF Pro2 mass spectrometer using a data‐independent acquisition strategy (12 × 50 m/z Window 4 IMS PASEF‐DIA method). Data files were searched against the Swissprot/Uniprot human protein database using the DIA‐NN 1.8.1 (Data‐Independent Acquisition by Neural Networks) software suite. Search settings were fixed modification for Cys carbamidomethylation, variable oxidation of methionine side chains, sample dependent mass accuracy, double pass mode for protein identification, precursor m/z 400–1100, fragment m/z 250–1700, report of protein identification quality and heuristic protein inference. Protein abundances were recovered from proteotypic peptides using an in‐house R‐script based on the one supplied by DIA‐NN. Sample statistics were performed in Perseus version 1.6.15.0 (Tyanova et al., 2016). Raw data have been deposited to the ProteomeXchange Consortium (Deutsch et al., 2023) via the PRIDE (Perez‐Riverol et al., 2022) partner repository with the dataset identifier PXD070244 (https://www.ebi.ac.uk/pride/archive/projects/PXD070244).

### 2.10 PMCA2 immunofluorescence microscopy and image processing

Plasma membrane calcium‐transporting ATPase 2 (PMCA2) immunofluorescence images were recorded using an Infinity Line system (Abberior Instruments GmbH, Göttingen, Germany) with a UPLXAPO60XO NA 1.42 objective and Imspector software version 16.3.16100 in LightBox mode. The confocal images were further deconvolved using Huygens Essential version 23.04.0p6 (Scientific Volume Imaging B.V., Hilversum, The Netherlands).

## 3 RESULTS

### 3.1 The spectrum of myopathological findings in young and aged healthy males

Cryo‐sections of the soleus muscle biopsy specimens from all 43 clinically healthy study participants were used for a myopathological evaluation based on hematoxylin and eosin (H&E), Oil red O, Gömöri trichrome (GT), combined cytochrome c oxidase/succinate dehydrogenase (COX/SDH), periodic acid Schiff (PAS), and myosin heavy chain (MHC) isoform staining (Table 1). This examiner‐based myopathological evaluation denoted the diagnosis of “normal skeletal muscle tissue” in 26 participants, in 10 out of 12 biopsies from young unathletic participants (Table 1, red; group hereafter referred to as “YUA”), in 9 out of 10 young power‐trained athletes (Table 1, blue; group hereafter “YPT”), in 7 out of 11 aged unathletic participants (Table 1, purple; group hereafter “AUA”), but in none of the 10 aged power‐trained athletes (Table 1, green; group hereafter “APT”). Three biopsies of the latter group and one more of AUA showed signs of chronic neurogenic atrophy defined by the presence of type I and type II fiber grouping along with atrophic fibers and nuclear clumps. APT had two more biopsies with pathological findings, one case of type II fiber atrophy defined by a large number of type II fibers with a >12% smaller fiber diameter compared to type I fibers, and one case of unspecific myositic changes defined by a small endomysial and small perimysial inflammatory infiltrate, groups of atrophic fibers, sporadic nuclear clumps, and an increased number of fibers with centralized nuclei. In an additional 11 biopsies (3 from young and 8 from aged participants) (Table 1), unspecific myopathological changes, defined by minor pathological alterations, were observed, which precluded the diagnosis of normal skeletal muscle.

**TABLE 1:** The spectrum of myopathological findings in the MALICoT.

Subjects		Key features from myological evaluation of H&E, oil red O, GT, COX/SDH, PAS, MHCs, MHCf, MHCd, MHCn stained M. Soleus sections								Diagnosis
		Pathological findings	Endomysial connective tissue	Oil red O pos. Droplets	Ragged‐red fibers	COX neg. Fibers	Type I fibers	Type II fibers	MHCd/n pos. Fibers	
BI5	Young and unathletic	Sporadic atrophic fibers	Slight increase	Middle‐sized	0	0	~70%–80%	~30%–40%	0	
BK1		None	Slight increase	Small‐sized	0	0	~80%–90%	~20%–30%	2	
BW3		Sporadic atrophic fibers	Slight increase	Middle‐sized	0	0	~60%	~40%–50%	5	
CQ1		Sporadic atrophic fibers	Normal	Small‐sized	0	0	~70%–80%	~30%–40%	0	
EJ9		Sporadic atrophic fibers	Normal	Middle‐sized	0	1	~80%	~30%	0	Unspec. myopathol. changes
GI3		One atrophic fiber	Slight increase	Small‐sized	0	0	~60%	~30%–40%	1	
GM5		Sporadic atrophic fibers	Slight increase	Small‐sized	0	1	~70%–80%	~30%–40%	3	
IF3		Sporadic atrophic fibers, one nucelar clump	Normal	Small‐sized	0	0	~70%–80%	~20%–30%	0	Unspec. myopathol. changes
KB5		Sporadic atrophic fibers	Slight increase	Middle‐sized	0	0	~80%–90%	~20%	1	
PN8		Two nucelar clumps	Slight increase	Middle‐sized	0	0	~80%–90%	~30%	0	
RL4		Sporadic nucelar clumps	Slight increase	Small‐sized	0	0	~80%–90%	~20%–30%	3	
SF6		Sporadic atrophic fibers	Normal	Small‐sized	0	0	~90%	~10%–20%	0	
DK8	Young and power‐trained athletes	Sporadic atrophic fibers	Slight increase	Small‐sized	0	0	~60%	~40%–50%	0	
GA5		One degenerating fiber, one atrophic fiber	Normal	Middle‐sized	0	0	~50%	~50%	2	
GY6		Sporadic atrophic fibers, two nucelar clumps	Slight increase	Small‐sized	0	0	~80%	~30%	0	
KO7		One degenerating fiber, multiple atrophic fibers, sporadic nuclear clumps	Slight increase	Small‐sized	0	0	~70%	~40%–50%	35	
PJ8		Sporadic atrophic fibers	Slight increase	Middle‐sized	0	0	~70%–80%	~20%–30%	0	Unspec. myopathol. changes
QC6		None	Slight increase	Middle‐sized	0	1	~70%–80%	~40%–50%	0	
QH5		None	Normal	Small‐sized	0	0	~80%	~30%	0	
RM9		Sporadic atrophic fibers	Normal	Small‐sized	0	0	~80%–90%	~30%–40%	0	
RP9		None	Slight increase	Small‐sized	0	0	~70%–80%	~30%–40%	2	
WL2		Sporadic atrophic fibers	Slight increase	Middle‐sized	0	0	~70%–80%	~20%–30%	13	
CC7	Aged and unathletic	Sporadic atrophic fibers	Slight increase	Small‐sized	0	0	~60%	~40%	8	Unspec. myopathol. changes
HA4		Sporadic atrophic fibers	Normal	Small‐sized	0	2	~70%	~30%–40%	4	Unspec. myopathol. changes
IK8		One nucelar clump	Normal	Small‐sized	0	2	~70%–80%	~20%–30%	1	
MS7		One degenerating fiber, multiple atrophic fibers, sporadic nuclear clumps, >3% fibers with centralized nuclei	Slight increase	Middle‐sized	4	1	~70%–80%	~30%–40%	39	Chronic neurogenic atrophy
QP4		Sporadic atrophic fibers	Normal	Small‐sized	0	0	~70%–80%	~30%	4	
RR9		Sporadic atrophic fibers, sporadic nuclear clumps	Slight increase	Small‐sized	0	0	~60%	~40%–50%	3	
TN6		Sporadic atrophic fibers, >3% fibers with centralized nuclei	Slight increase	Small‐sized	1	3	~60%	~40%–50%	0	Unspec. myopathol. changes
UI2		Sporadic atrophic fibers, sporadic nuclear clumps	Slight increase	Small‐sized	0	3	~90%	~30%–40%	0	
VP2		Sporadic atrophic fibers, sporadic nuclear clumps	Normal	Middle‐sized	0	1	~90%	~20%–30%	12	
XF8		Sporadic nuclear clumps	Normal	Small‐sized	0	1	~70%–80%	~40%–50%	11	
YQ2		Sporadic atrophic fibers, sporadic nuclear clumps	Slight increase	Small‐sized	0	5	~80%–90%	~20%–30%	3	
DP4	Aged and power‐trained athletes	Sporadic atrophic fibers	Slight increase	Small‐sized	1	3	~80%	~40%	12	Unspec. myopathol. changes
DY6		Sporadic regenerating fibers, sporadic atrophic fibers, sporadic nuclear clumps	Slight increase	Small‐sized	1	0	~70%–80%	~30–40%	10	Chronic neurogenic atrophy
ED5		One endomysial and one perimysial inflammatory infiltrate, groups of atrophic fibers, sporadic nuclear clumps, >3% fibers with centralized nuclei	Slight increase	Coarse‐sized	3	2	~90%	~10%	18	Unspec. myositic changes
FP7		One regenerating fiber, sporadic atrophic fibers	Slight increase	Coarse‐sized	0	0	~100%	~10%	7	Beginning type II fiber atrophy
HR9		Two atrophic fibers, two nuclear clumps	Slight increase	Small‐sized	0	0	~90%	~15%	10	Unspec. myopathol. changes
LP1		Sporadic atrophic fibers	Slight increase	Small‐sized	1	0	~90%–100%	~10%–20%	14	Unspec. myopathol. changes
OY5		One regenerating fiber, sporadic nuclear clumps	Normal	Small‐sized	0	0	~80%	~30%–40%	7	Chronic neurogenic atrophy
VO8		Sporadic atrophic fibers, one nuclear clump	Slight increase	Small‐sized	0	0	~90%	~10%	16	Unspec. myopathol. changes
XV3		One regenerating fiber, groups of atrophic fibers, sporadic nuclear clumps	Slight increase	Small‐sized	0	0	~90%	~20%	18	Chronic neurogenic atrophy
YB2		Sporadic atrophic fibers	Normal	Small‐sized	0	0	~90%	~10%	0	Unspec. myopathol. changes

*Note*: The Master Athletic Laboratory Study of Intramuscular Connective Tissue (MALICoT, registration number DRKS00015764) included a total of 43 participants, divided into 12 young (20 to 35 years) unathletic participants, 10 young power‐trained athletes, 11 aged (60 to 75 years) unathletic participants and 10 aged power‐trained athletes. The alpha‐numeric subject IDs are randomly generated combinations of two letters and a digit. Myopathological evaluation was based on H&E, Oil red O, Gömöri trichrome (GT), COX/SDH, PAS, slow MHC, fast MHC, developmental MHC and neonatal MHC stained cryo‐sections of soleus muscle biopsy specimens. Pathological findings, specific myopathological findings; endomysial connective tissue, endomysial thickness estimated in the H&E‐stained section, normal or slightly increased; Oil red O‐positive droplets, classification as small‐, middle‐, or coarse‐sized; ragged‐red fibers, number of fibers containing clumps of mitochondria in the GT‐stained section; COX‐negative fibers, number of COX‐negative fibers counted in the COX/SDH double‐stained fibers; type I and type II fibers, estimated fractions in percent of fibers positive for either slow or fast MHC isoforms, fractions were determined in separately stained sections and therefore do not necessarily add up to 100%; MHCd/n positive fibers, total number of positive fibers from sections stained separately for developmental or neonatal MHC isoform expression; diagnosis, myopathological diagnosis, if present.

### 3.2 Conventional and AI‐based quantitation of endomysium content and mean fiber cross‐sectional area

**FIGURE 1:**
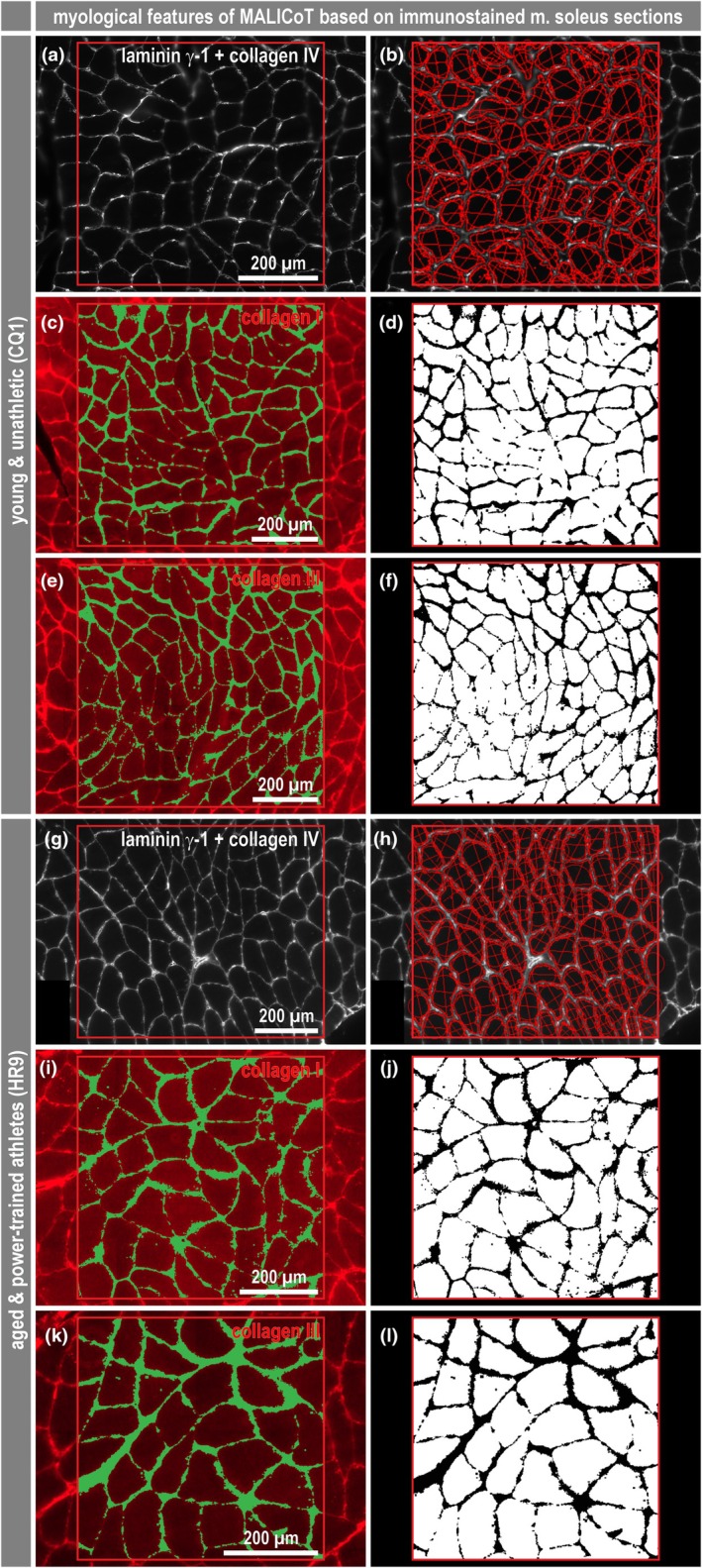
Myological parameters of MALICoT (I): Immunostained sections of soleus muscle and manual digital image analysis. Exemplary images of soleus muscle sections from a young unathletic participant (A, C, E) and an aged athletic participant (G, I, K) double‐immunostained for laminin‐γ‐1/collagen IV (A, G), and immunostained for either collagen I (C, I) or collagen III (E, K). The red squares indicate ROIs selected for segmentation of muscle fibers and endomysium using the microscope software ZEN. In the case of laminin‐γ‐1/collagen IV staining this analysis resulted in an approximation of individual fiber areas and Feret diameters (B, H) and a calculated determination of the endomysium. For collagen I and collagen III staining, the number of muscle fibers was counted and image analysis resulted in black and white images to determine the fiber (sum of white pixels) and endomysium (sum of black pixels) areas (D, F, J, L). Further details on analysis and calculation of derived parameters are provided in the Materials and Methods section.

**FIGURE 2:**
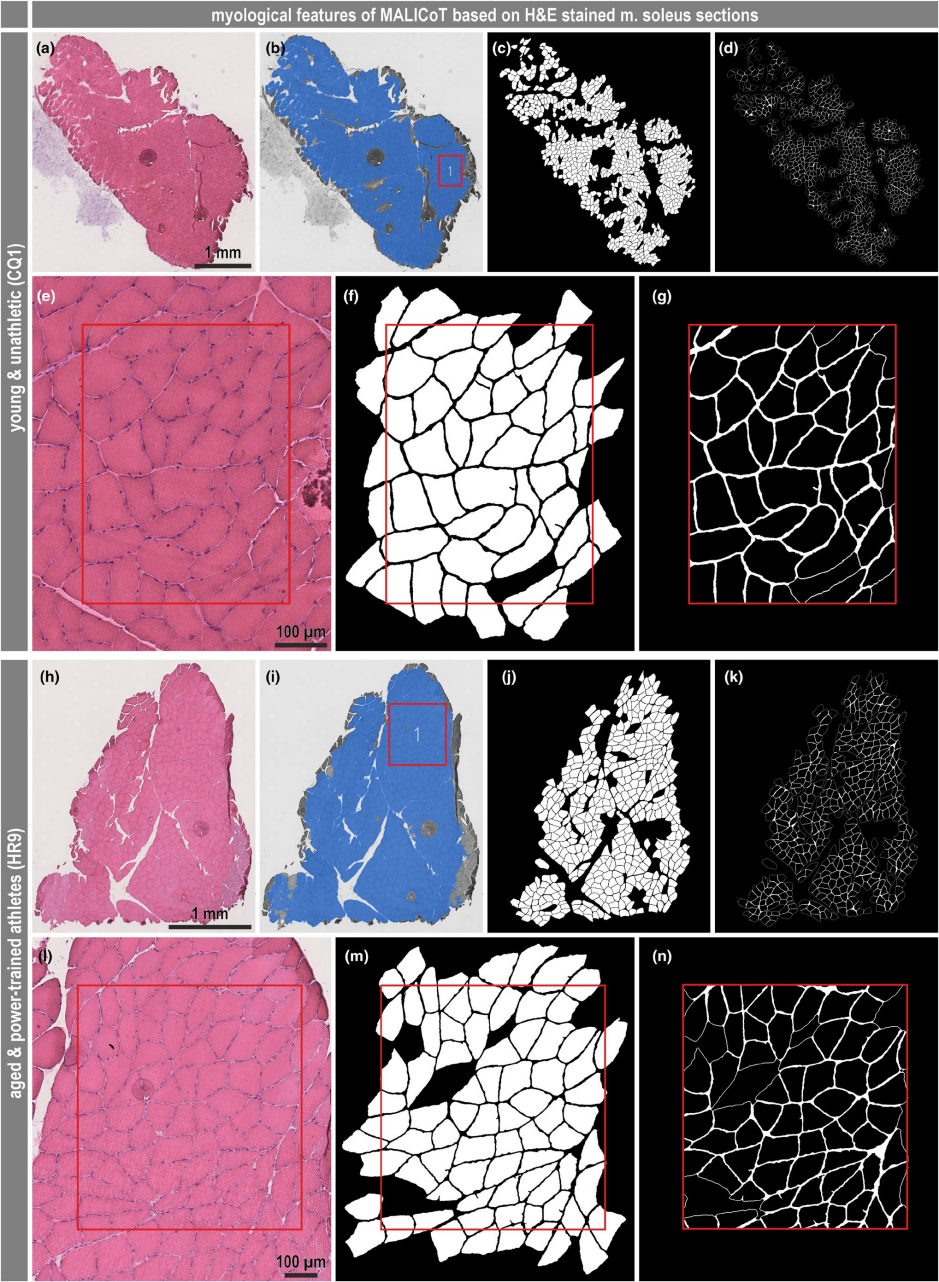
Myological parameters of MALICoT (II): H&E‐stained sections of soleus muscle and automated deep learning‐based image analysis. Exemplary images of H&E‐stained soleus muscle sections from the same young unathletic (A, E) and aged athletic (H, L) participants as in Figure 1. Muscle fibers (C, F, J, M) and endomysium (D, G, K, N) were automatically segmented for the whole sections (A–D, H–K) or ROI‐based (red squares) (E–G, L–N). For whole section analysis, the sections and artifacts were automatically detected prior to segmentation (B, I), and areas of the sections that passed quality control parameters are shown in blue; for the ROI‐based analysis, the ROI was manually set as in Figure 1. Further details on image analysis and the quantitative parameters obtained are provided in the Materials and Methods section.

To address the possible presence of more subtle changes in the amount or thickness of the endomysial connective tissue, different quantitative approaches were used to measure the endomysial content. Double immunofluorescence staining for laminin‐γ‐1 and collagen IV in transversal cryo‐sections, as described previously (Thot et al., 2021), was used to indirectly determine the endomysial content based on the lines of laminin‐γ‐1‐ and collagen IV‐positive basal laminae and the nonstained stripe in between (Figure 1a,b,g,h). In addition, collagen I (Figure 1c,d,i,j) and collagen III (Figure 1e,f,k,l) single immunofluorescence staining was used to directly visualize the connective tissue between the muscle fibers. As a new approach, an automated deep learning and photorealistic computer graphics generated synthetic data based artificial intelligence analysis tool (Mill et al., 2025) was used on images of the H&E‐stained sections (Figure 2e–g,l–n). All analysis approaches were performed in a region of interest (ROI)‐based manner, with the latter approach additionally performed as a full‐sample analysis on whole slide images (Figure 2a–d,h–k). The immunofluorescence approaches (Figure 1) required an optimized staining protocol with less unspecific background and tissue autofluorescence signal and several steps of semi‐automatic and manual image analysis. In contrast, the automated deep learning‐based approach (Figure 2) was not limited to a region of interest but was also used to analyze whole slide images containing multiple H&E‐stained sections. Furthermore, this software tool directly segmented individual muscle fibers and also the surrounding endomysium.

### 3.3 Young power‐trained athletes and aged unathletic participants compared to young unathletic participants: Elevated mean fiber cross‐sectional area, but no difference in endomysium content

**FIGURE 3:**
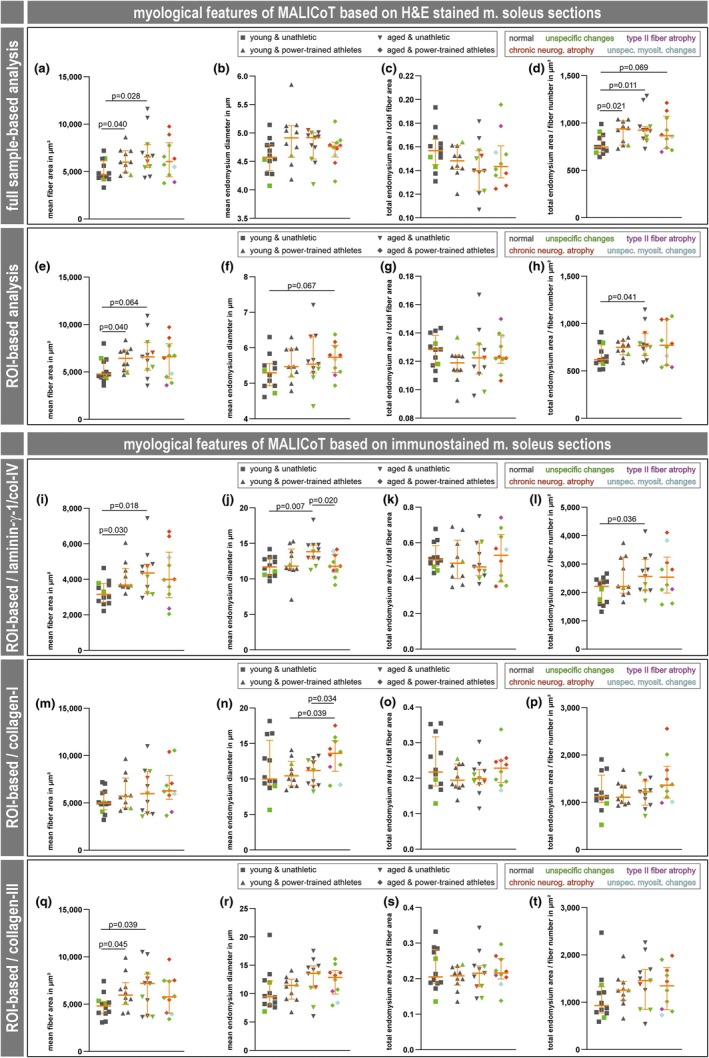
Compilation of myological parameters of MALICoT obtained through automated deep learning‐based and manual digital image analysis of soleus muscle sections. Four main myological parameters are shown, that is, mean fiber area in μm^2^, mean endomysium thickness in μm, total endomysium area/total fiber area, and total endomysium area/fiber number in μm^2^, for each of the five different digital image analysis procedures, that is, H&E‐stained full sample and ROI‐based automated deep learning (rows 1 and 2) and fluorescence‐stained extracellular matrix component ROI‐based manual digital analysis (rows 3 to 5). In the case of automated analysis, the values for fiber area, endomysium thickness, total endomysium area and fiber number were obtained directly from the analyzed images; in the case of the manual analysis, the values for the four parameters shown were derived from other primary parameters, depending on the type of manual analysis, from calculations using the values for total fiber area, total number of fibers, number of intact fibers, number of trimmed fibers, ROI dimensions, total fiber perimeter, or total endomysium area; details are provided in the Materials and Methods section. Each graph shows a scatter plot (young unathletic *n* = 12, young power‐trained athletic *n* = 10, aged unathletic *n* = 11, aged power‐trained athletic *n* = 10) with median and interquartile range (in orange). After normality testing, individual statistical significances were calculated using unpaired two‐sample Welch's *t*‐tests, except for comparisons involving mean fiber area of young power‐trained athletes from laminin/collagen IV staining, which were done using the Mann–Whitney *U* test, as this dataset did not have a normal distribution. Dark gray symbols, normal skeletal muscle; green symbols, unspecific myopathological changes; red symbols, chronic neurogenic atrophy; magenta symbols, type II fiber atrophy; turquoise symbols, unspecific myositic changes; see Results section for details.

The data obtained using the different quantification approaches showed similar overall results for the four main myological parameters shown, that is, mean fiber area in μm^2^, mean endomysium thickness in μm, total endomysium area/total fiber area, and total endomysium area/fiber number in μm^2^ (Figure 3). Deep learning‐based analysis of H&E‐stained soleus muscle sections in whole slide scans showed significantly higher levels of (i) the mean fiber area when comparing YPT with YUA, and AUA with YUA (Figure 3a) and (ii) the total endomysium area/fiber number ratio when comparing YPT with YUA, AUA with YUA, and APT with YUA (Figure 3d). The same approach produced very similar patterns in the scatter plots derived from the ROI‐based analysis, where significantly higher levels were detected for (i) the mean fiber area when comparing YPT with YAU, and AUA with YUA (Figure 3e), (ii) the mean endomysium thickness when comparing APT with YUA (Figure 3f), and (iii) the total endomysium area/fiber number ratio when comparing AUA with YUA (Figure 3h). ROI‐based analyses of the immunostained soleus muscle sections showed significantly higher levels (i) in the case of laminin‐γ‐1/collagen IV and collagen III staining for the mean fiber area when comparing YPT with YAU, and AUA with YUA (Figure 3i,q), (ii) in the case of laminin‐γ‐1/collagen IV staining for the mean endomysium thickness and the total endomysium area/fiber number ratio when comparing AUA with YUA (Figure 3j,l), and (iii) in the case of collagen I for the mean endomysium thickness when comparing APT with YPT (Figure 3n). Two other significant findings should be considered with caution, the lower value for the mean endomysium thickness in the case of laminin‐γ‐1/collagen IV staining when comparing APT with AUA (Figure 3j) is contradicted by the collagen I staining showing a higher value for this comparison (Figure 3n).

**FIGURE 4:**
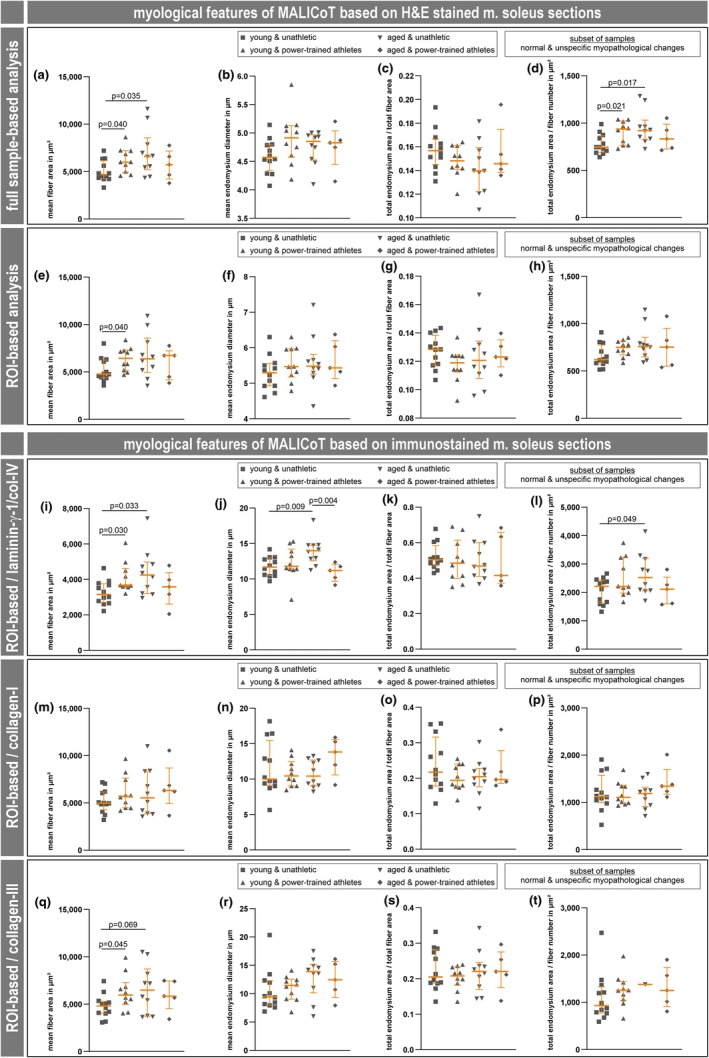
Adjusted MALICoT dataset excluding biopsies with distinct myopathological diagnoses. Four main myological parameters are shown, that is, mean fiber area in μm^2^, mean endomysium thickness in μm, total endomysium area/total fiber area, and total endomysium area/fiber number in μm^2^, for each of the five different digital image analysis procedures, that is, H&E‐stained full sample and ROI‐based automated deep learning (rows 1 and 2) and fluorescence‐stained extracellular matrix component ROI‐based manual digital analysis (rows 3 to 5). In contrast to Figure 3, the compilation of myological parameters in this figure is based on the adjusted MALICoT dataset excluding biopsies with distinct myopathological diagnoses. Each graph shows a scatter plot (young unathletic *n* = 12, young power‐trained athletic *n* = 10, aged unathletic *n* = 10, aged power‐trained athletic *n* = 5) with median and interquartile range (in orange); the dark gray symbols in the scatter plots include both the biopsies with normal skeletal muscle and with unspecific myopathological changes. See the legend of Figure 3 for additional details.

To improve accuracy, this quantification data obtained from the 43 clinically healthy participants was also once adjusted for the biopsies with distinct myopathological diagnoses, i.e., only data from biopsies that were deemed to be normal or only exhibited unspecific myopathic changes was considered (Figure 4). This reduced the number of biopsies from AUA by one into *n* = 10 and halved the number of biopsies from APT into *n* = 5. Although the overall pattern of the scatter plots still remained very similar (Figure 4), six group‐specific comparisons lost statistical significance. When analyzing the H&E‐stained soleus muscle sections in whole slide scans significantly higher levels remained for the mean fiber area as well as the total endomysium area/fiber number ratio when comparing YPT with YUA, and AUA with YUA (Figure 4a,d). The ROI‐based analysis of H&E‐stained sections solely showed a significantly higher level for the mean fiber area when comparing YPT with YUA (Figure 4e). While there was no difference in the ROI‐based analysis in the case of the laminin‐γ‐1/collagen IV‐ and collagen III‐stained soleus muscle sections (Figure 4i,j,l,q), significant effects in the collagen I based analysis were lost (Figure 4n).

In summary, the following effects were consistent and statistically significant: (1) A markedly higher level of the mean fiber cross‐sectional area when comparing YPT with YUA (6189 ± 1298 μm vs. 5026 ± 1128 μm, mean ± standard deviation, Figure 4a). (2) An even higher level of the mean fiber cross‐sectional area when comparing AUA with YUA (7011 ± 2441 μm vs. 5026 ± 1128 μm, Figure 4a). (3) Analogously higher levels of the ratio of total endomysium area to fiber number when comparing YPT with YUA (901 ± 122 μm vs. 777 ± 105 μm) and AUA with YUA (952 ± 180 μm vs. 777 ± 105 μm) (Figure 4d). Notably, no consistent, significant changes were found for the endomysium thickness or the ratio of total endomysium area to total fiber area.

### 3.4 Proteomic analysis revealed less abundant plasma membrane calcium‐transporting ATPase 2 in soleus muscle from aged power‐trained athletes

**FIGURE 5:**
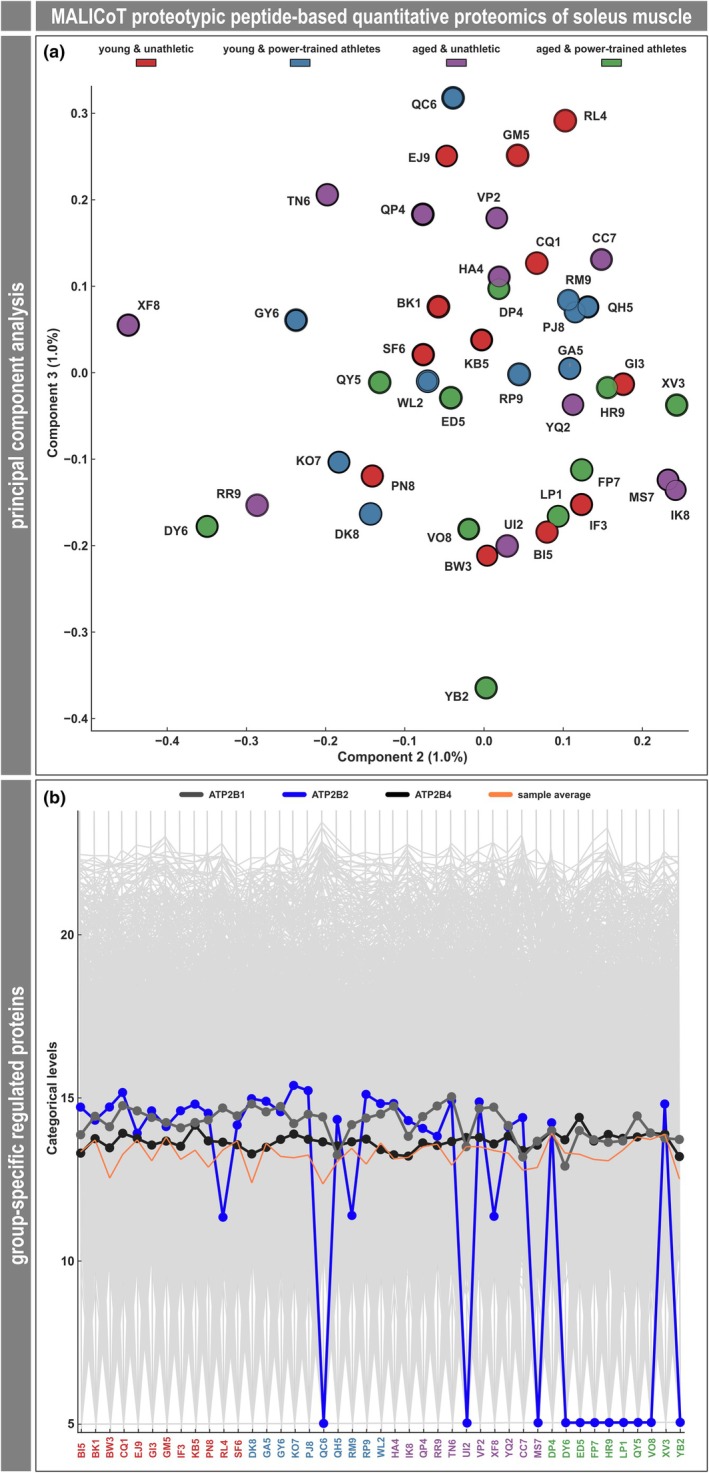
Proteotypic peptide‐based quantitative proteomic analysis of MALICoT‐derived soleus muscle tissue. (a) Based on the quantitation of 3954 proteins, principal component analysis (PCA) determined that there were no major differences in the global protein expression pattern. (b) Manual inspection of several muscle and connective tissue‐related proteins revealed similar abundances in all samples. However, one of the quantitated proteins, plasma membrane calcium‐transporting ATPase 2 (ATP2B2), was not detected in 8 out of the 10 samples from the aged power‐trained athletes. Color code as in Table 1 and Table S1.

In a next step, soleus muscle tissue from the 43 MALICoT participants was used for proteomic analysis. Based on proteotypic peptides, a number of 3954 proteins was quantified and their overall abundance distribution was uniform across all samples. Principal component analysis (PCA) revealed a picture of 43 individual proteomes showing no grouping in terms of the MALICoT participant classification (Figure 5a). Similarly, heat‐map plots from hierarchical clustering showed no clustering according to the four MALICoT groups or any other clustering of the samples at all (not shown). Despite this finding that there was no grouping, further standard data analysis steps were performed. Filtering of the 3954 proteins to include only those with ≥70% valid values in at least one of the four groups resulted in 3615 quantified proteins (Table S1, 1st tab). ANOVA with permutation‐based FDR (0.05) identified 24 proteins with significantly different abundances among the four MALICoT groups (Table S1, 1st tab, column R). However, even at this level, hierarchical cluster analysis revealed clustering to only a very limited extent, notably not matching the MALICoT groups (Table S1, 2nd tab). A small cluster comprising 5 out of the 10 APT samples (Table S1, 3rd tab) did not match the 5 biopsies, which in this group showed only the unspecific myopathic changes (see Table 1, Figure 4). Subsequent multiple‐testing corrected (FDR 0.05) two‐sample Welch's *t*‐tests revealed the significant differences in the abundances of 14 proteins comparing YUA vs. APT, of 20 proteins comparing YPT vs. APT, and of 2 proteins comparing YPT vs. AUA (Table S1, 1st tab, columns AD, AL, AH, respectively). Venn analysis demonstrated no intersection between the protein lists of these three comparisons. In the intersection of YUA vs. APT, and YPT vs. APT, Venn analysis highlighted 12 proteins (Table S1, 4th tab). It is important to note at this point that when the results from ANOVA, Welch's *t*‐tests, and Venn analysis are considered together, no proteins remain that show a statistically significant difference in abundance solely due to power‐trained or age. Moreover, GOBP, GOMF, GOCC, KEGG BRITE, and Reactome name annotations in conjunction with 1D‐enrichment analysis did not identify any significantly enriched groups of proteins.

**FIGURE 6:**
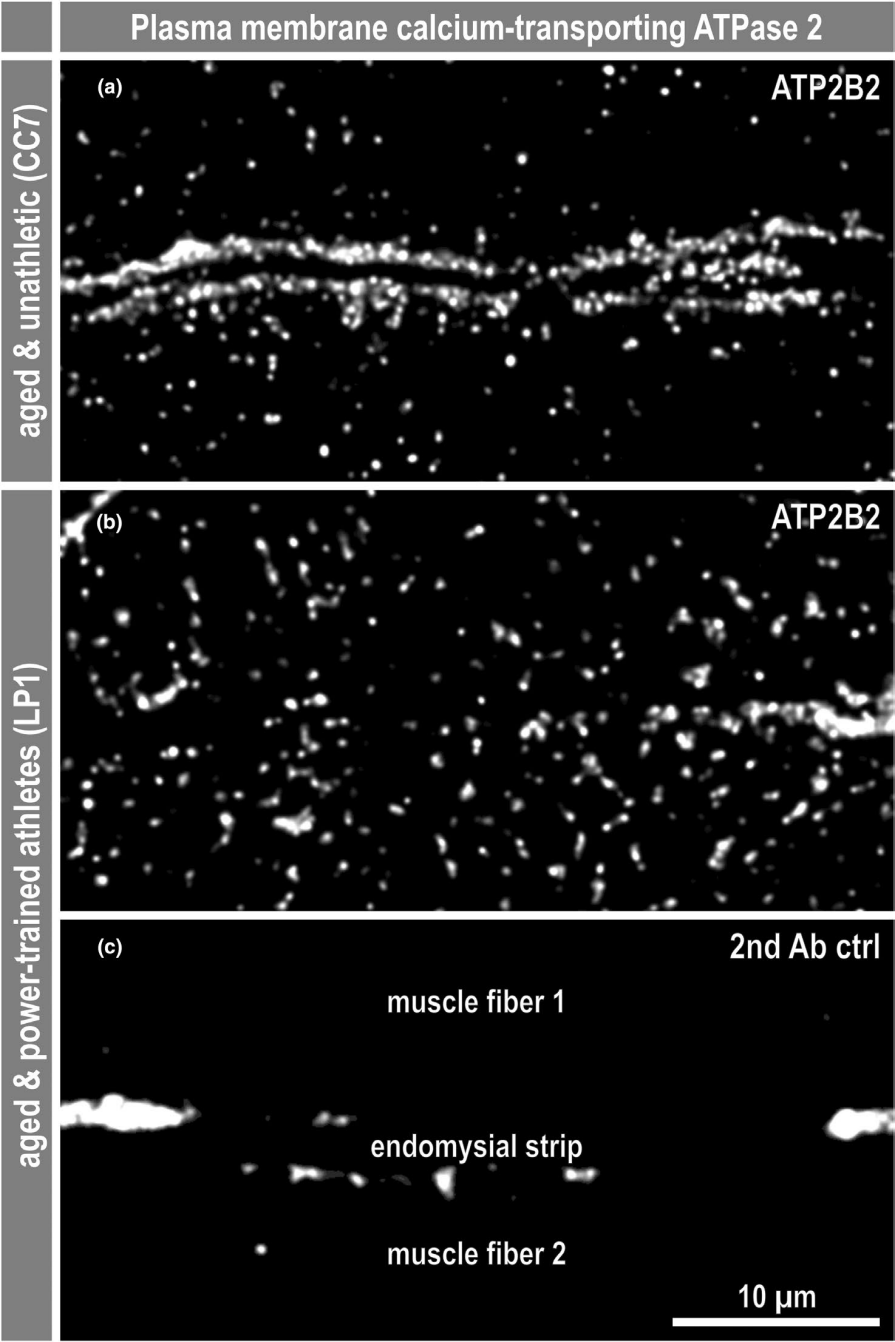
Aged power‐trained athletes have less plasma membrane calcium‐transporting ATPase 2 at the sarcolemma. To verify the proteomic finding of a reduction of plasma membrane calcium‐transporting ATPase 2 (ATP2B2 or PMCA2) below the detection limit specifically in the aged power‐trained athletes, samples from aged unathletic (HA4, TN6, YQ2, CC7) and aged power‐trained athletes (HR9, LP1, VO8, YB2), who had no specific diagnosis (Table 1), were immunostained for ATP2B2. (a) As expected, the subcellular distribution of ATP2B2 in the aged unathletic samples appeared as strings of spots in a row at the level of the two sarcolemmas in the center of the field of view. (b) In contrast, there was no signal enrichment at the sarcolemma in samples from aged power‐trained athletes, but only single spots in the sarcoplasm. (c) Negative controls with secondary antibody only show few artifacts from tissue autofluorescence and no ATP2B2‐specific signals. All three images show a section of sarcoplasm from one muscle fiber, the endomysium in the center of the image, and a section of an adjacent muscle fiber below. The confocal images were deconvolved using Huygens Essential (Scientific Volume Imaging B.V., Hilversum, The Netherlands).

Additional manual inspection of the data confirmed that there were no differences in the abundances of any of the detected collagens, laminins, nidogens, or other proteins related to the composition of intramuscular connective tissue, such as dermatopontin, fibrillin‐1, and perlecan (basement membrane‐specific heparan sulfate proteoglycan core protein) (Table S1, 1st tab). However, in a proteomic dataset, changes for which no fold change can be calculated may also be of interest. Examining the dataset for proteins that were detected in some samples but below the detection limit (or not expressed at all) in other samples from the MALICoT groups led to the identification of a single candidate of interest, plasma membrane calcium‐transporting ATPase 2 (ATP2B2 or PMCA2). While plasma membrane calcium‐transporting ATPases 1 and 4 (ATP2B1 and ATP2B4) were detected in all samples from all four MALICoT groups, ATP2B2 was detected in 12 out of 12 samples from YUAs, in 9 out of 10 from YPT (not detected in sample QC6) and in 9 out of 11 from AUA (not detected in samples MS7 and UI2), but only in 2 (samples DP4 and XV3) out of 10 from APT (Figure 5b; Table S1, 1st tab, columns L‐O). In this respect, imputing missing values as an additional step between the above‐mentioned filtering of valid values and ANOVA caused the absence pattern of ATP2B2 to appear as a significantly different abundance (Table S1, 5th tab). To verify this marked difference in the abundance of ATP2B2, the proteomic analysis was complemented by ATP2B2 immunofluorescence imaging of samples from AUA and APT. In contrast to a spot chain‐like sarcolemmal localisation of the ATP2B2 signals in the samples from AUA, no such signal enrichment was found at the sarcolemma in samples from APT (Figure 6).

## 4 DISCUSSION

The cross‐sectional Master Athletic Laboratory Study of Intramuscular Connective Tissue (MALICoT, DRKS00015764) primarily set out to analyze effects of athletic exercise and age on the endomysium content in the human soleus muscle. Based on previously published results, it was assumed that age (Alnaqeeb et al., 1984; Fede et al., 2022; Yeung et al., 2023) and low physical activity (Thot et al., 2021) might lead to increased endomysium content. To the best of our knowledge, no studies addressing endomysium content as a function of age and training state have been published in humans. Comparing Masters athletes with sedentary individuals at different ages thus allows us to study age‐related muscle changes without the confounding factor of sedentary behavior (Rittweger et al., 2004). Another key aspect of this work was to compare our previously published approach of quantifying endomysium content and muscle fiber size using laminin‐γ‐1 immunostaining of muscle tissue cryosections (Thot et al., 2021), in this work conducted as laminin‐γ‐1/collagen IV double‐immunostaining, with collagen I and collagen III immunostainings, as well as deep learning‐based artificial intelligence analysis of H&E‐stained cryosections (Mill et al., 2025).

### 4.1 Deep learning‐based analysis of H&E‐stained muscle sections is a new state of the art technique

While laminin‐γ‐1 and collagen IV antibodies stain the basement membrane and delineate the endomysial space, allowing segmentation of muscle fibers in immunofluorescence images (Figure 1a,b,g,h), the endomysium content must be calculated (Thot et al., 2021). Conversely, when using antibodies directed against collagen I and collagen III, the endomysium area is labeled (Figure 1c–f,i–l) and the muscle fiber cross‐sectional area must be derived. All immunostaining variants require calculation, and to a certain extent the estimation, of fiber area and endomysium thickness. To do so, manual work is required with regard to immunofluorescence staining protocols, immunofluorescence microscopy and image processing. In contrast, the deep learning‐based image analysis only requires standard H&E‐stained muscle tissue cross‐sections and can also work with whole slide images containing multiple stained sections acquired by a slide scanner (Figure 2). Most importantly, this approach obtains pixel‐accurate values of fiber area and endomysium thickness from ROIs or single and multiple sections (Mill et al., 2025).

**FIGURE 7:**
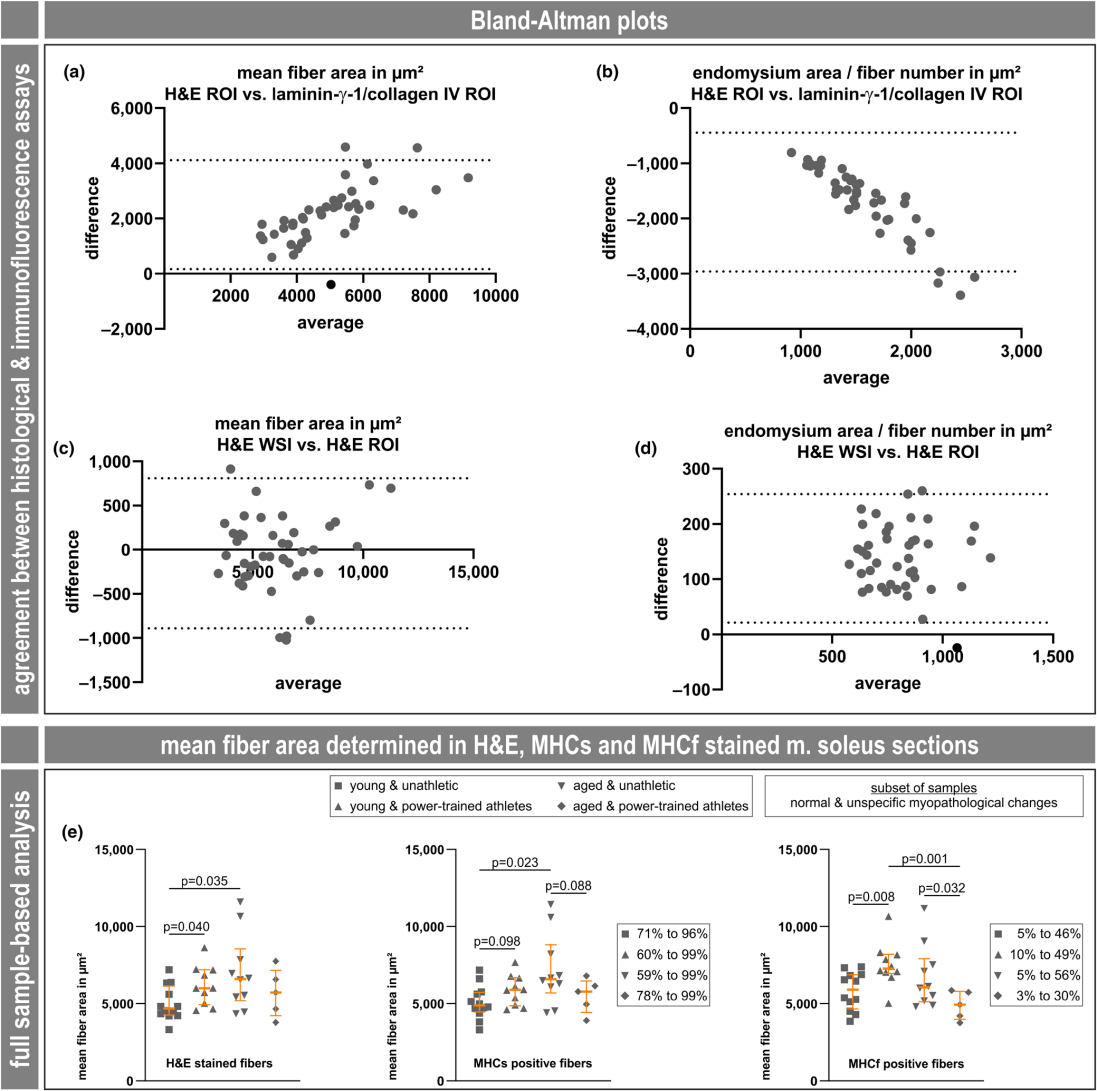
Bland–Altman plots of agreement between analysis approaches, and attribution of the significantly increased mean fiber area to the fiber type. (a–d) Bland–Altman plots of mean fiber area and total endomysium area to fiber number ratio for ROI‐based analysis of H&E‐stained and laminin‐γ‐1/collagen IV immunostained sections as well as of whole slide scan‐based and ROI‐based analyses of H&E‐stained soleus muscle sections. (E) Scatter plots with median and interquartile range (in orange) of the mean fiber area in μm^2^ derived from full sample automated deep learning analysis of H&E (same graph as shown in Figure 4a), and MHCs and MHCf stains, limited to the diagnoses‐adjusted MALICoT dataset. For MHCs and MHCf, the range of positively stained fiber fractions as a percentage is indicated.

Overall, the scatter plot patterns (Figure 3) appeared similar and consistently showed statistical significance between groups for the mean fiber cross‐sectional area and the total endomysium area to fiber number ratio obtained by the deep learning‐based analysis (Figure 3A–H), as well as for the laminin‐γ‐1/collagen IV and collagen III immunostains (Figure 3I–L, Q–T). Bland–Altman plots of the two parameters, mean fiber area and total endomysium area to fiber number ratio, revealed agreement between ROI‐based analysis of H&E‐stained and laminin‐γ‐1/collagen IV immunostained sections. In the case of mean fiber area, there was positive simple proportional bias (Figure 7a), and in the case of total endomysium area to fiber number ratio there was negative simple proportional bias (Figure 7b). Additional Bland–Altman plots showed consistency between the whole slide scan‐based and the ROI‐based analyses of H&E‐stained soleus muscle sections. In the case of mean fiber area, there was close to zero mean difference (Figure 7c), and in the case of total endomysium area to fiber number ratio, there was positive mean difference (Figure 7d). The latter can be explained by the fact that the ROIs were set to regions of the muscle cross‐sections that did not contain perimysium. Since the segmentation masks derived from the deep learning‐based analysis precisely fit the H&E staining pattern (Mill et al., 2025; and this work), and the Bland–Altman plots demonstrate that the deep learning‐based analysis of H&E‐stained muscle sections is an appropriate and simple substitute for the immunostaining approaches, this approach should be considered state of the art technique.

### 4.2 The states of “power‐trained” and “aged” have no significant impact on the endomysium content of human soleus muscle

In rat soleus and extensor digitorum longus muscles, a marked increase in endomysium and collagen content with age has been reported, which correlated with increased muscle stiffness (Alnaqeeb et al., 1984). In mouse lateral gastrocnemius muscle, it was found that the abundance of multiple extracellular matrix proteins in the intramuscular connective tissue increased with age, while wheel running exercise had no effect (Yeung et al., 2023). In human vastus lateralis muscle, endomysium content and collagen staining intensities were basically similar between endurance‐trained athletes and untrained controls (Mackey et al., 2005). In general, it seems that there is little published data on the relationship between intramuscular connective tissue or endomysium content and physical activity or age, and the results are contradictory.

Based on the full histological MALICoT data set (Figure 3) in conjunction with the adjusted dataset that excluded biopsies with distinct myopathological diagnoses (Figure 4), neither the mean endomysium thickness (Figure 4b) nor the ratio of endomysium to muscle fiber area (Figure 4c) were significantly affected by the training state or age. Only the ratio of the endomysium area to fiber number showed a higher value when comparing the groups of young power‐trained athletes with young unathletic participants and aged unathletic participants with young unathletic participants (Figure 4d). The additional proteomic analysis also revealed no significant differences in the abundances of proteins related to the composition of intramuscular connective tissue (Table S1, 1st tab). The only proteomic finding, which was also confirmed by immunofluorescence analysis, was a markedly lower abundance of plasma membrane calcium‐transporting ATPase 2 (ATP2B2 or PMCA2) in the group of aged power‐trained athletes (Figures 5b, 6). No data is currently available on the function of this ATP‐dependent Ca^2+^ pump isoform in skeletal muscle cells (Stauffer et al., 1993, 1994). ATP2B2 (UniProt entry Q01814) may be involved in regulating the basal sarcoplasmic Ca^2+^ concentration or transporting Ca^2+^ to the endomysial space.

### 4.3 The “power‐trained” and “aged” states were associated with a significantly higher fiber cross‐sectional area in human soleus muscle

The complete and the diagnoses‐adjusted MALICoT datasets showed a significantly higher level of the mean fiber cross‐sectional area when comparing young power‐trained athletes with young unathletic participants, and even more prominent when comparing aged unathletic participants with young unathletic participants (Figures 3a, 4a). The finding of an elevated mean fiber area in power‐trained human muscle is well‐documented and confirms previously reported findings (for example, Häggmark et al., 1978; Hermansen & Wachtlova, 1971). However, the influence of age on fiber cross‐sectional area in human muscle is apparently complex and inconsistent. Here, a meta‐analysis including 19 different studies on male vastus lateralis muscle and 1 on male gastrocnemius muscle (Figure 3 and table 3 in reference (Lee et al., 2024)) revealed that the fiber cross‐sectional area can markedly decrease, remain approximately the same, or increase markedly with age. The few studies on female muscles included in this meta‐analysis showed the same pattern (Lee et al., 2024). To include the fiber type in our finding of a significantly higher level of the fiber cross‐sectional area, we additionally analyzed the MHCs‐ and MHCf‐stained sections using the deep learning‐based image analysis software. In the diagnoses‐adjusted MALICoT data, the soleus muscle contained a high proportion of MHCs‐positive type I fibers (between 59% and 99%, as expected) and lower amounts of MHCf‐positive type II fibers (between 3% and 56%) (Figure 7e). For both fiber types, the patterns of mean fiber area differences between the four groups were basically the same as that detected by H&E staining; however, with different levels of statistical significance (Figure 7e). In this respect, MALICoT adds first data on human male soleus muscle.

Notably, significant differences in the fiber area (or “fiber cross‐sectional area,” not to be confused with “muscle cross‐sectional area”) and the fact that muscle tissue specimens of essentially constant dimensions are analyzed by means of a muscle biopsy inevitably result in differences in values referring to the fiber number. In the case of MALICoT, the significantly larger fiber area associated with a lower fiber number in the cross‐sections of the biopsy specimens can explain the observed elevation in the endomysium area‐to‐fiber‐number ratio. Therefore, the difference in this relative value should be disregarded as an artifact.

In summary, MALICoT, with evidence from a group size of 10 to 12 male participants, showed as primary result that athletic exercise and age did not significantly affect thickness or area of the endomysium, or its protein composition in the human soleus muscle. Additionally, this study revealed that the fiber cross‐sectional area in the human soleus muscle was significantly elevated in the groups of young power‐trained athletes and aged unathletic participants. Since 17 of the 43 clinically healthy study participants exhibited relevant histopathological findings, a detailed myopathological analysis seems necessary in studies examining effects of exercise, age, or other factors on muscle, even when asymptomatic participants were recruited. Furthermore, the quantitative proteomic analysis in this study suggests that a larger sample size is required when analyzing human muscle tissue than has been established when studying muscle tissue from inbred mouse strains, for example, in order to obtain significant results on the biochemical level.

## AUTHOR CONTRIBUTIONS

**Christoph S. Clemen:** Conceptualization; data curation; formal analysis; investigation; methodology; project administration; resources; supervision; validation; visualization. **Sebastian W. Humbsch:** Formal analysis; investigation; methodology; visualization. **Carolin Berwanger:** Formal analysis; investigation; methodology; visualization. **Leonid Mill:** Formal analysis; methodology; software. **Andreas Schmidt:** Formal analysis; methodology; resources; visualization. **Rolf Schröder:** Conceptualization; formal analysis; methodology; resources; validation. **Jörn Rittweger:** Conceptualization; data curation; formal analysis; investigation; methodology; project administration; resources; supervision; validation.

## FUNDING INFORMATION

This research received no specific grant from any funding agency in the public, commercial, or not‐for‐profit sectors. The study was funded with internal funding from the German Aerospace Centre (DLR), Department of Muscle and Bone Metabolism.

## CONFLICT OF INTEREST STATEMENT

The authors declare that they have no competing interests. C.S.C. and R.S. serve as consultants for MIRA Vision Microscopy GmbH.

## ETHICAL APPROVAL

This study was approved by the Ethics Committee of the North Rhine Medical Association in Düsseldorf (#2018269), Germany, and registered in the German Clinical Trials Register (DRKS‐ID DRKS00015764). All participants gave written informed consent before participating in the study.

## Supporting information


**Table S1.** Results of the proteome analysis. Dataset from proteotypic peptide‐based quantitative proteomic analysis using soleus muscle tissue samples from the 43 MALICoT participants. Out of 3954 quantified proteins, 3615 remained after filtering those with ≥70% valid values in at least one of the four MALICoT groups (1st tab). Considering the results of ANOVA, Welch's *t*‐tests, and Venn analysis, no proteins remain that show a statistically significant difference in abundance solely due to “power‐trained” or “age”. GOBP, GOMF, GOCC, KEGG BRITE, and Reactome name annotations in conjunction with 1D‐enrichment analysis did not identify any significantly enriched groups of proteins. For further details as well as on tabs 2–5 please refer to the Results section.

## Data Availability

Proteomic raw data have been deposited to the ProteomeXchange Consortium (Deutsch et al., 2023) via the PRIDE (Perez‐Riverol et al., 2022) partner repository with the dataset identifier PXD070244. Anonymized subject data used and analyzed for this report will be shared upon reasonable request.
